# Muscle magnetic resonance imaging involvement patterns in nemaline myopathies

**DOI:** 10.1002/acn3.51816

**Published:** 2023-06-02

**Authors:** Luke Perry, Georgia Stimpson, Leeha Singh, Jasper M. Morrow, Sachit Shah, Giovanni Baranello, Francesco Muntoni, Anna Sarkozy

**Affiliations:** ^1^ The Dubowitz Neuromuscular Centre, MRC Centre for Neuromuscular Diseases, Neurosciences Unit Great Ormond Street, Institute of Child Health, Great Ormond Street Hospital, University College London London UK; ^2^ NIHR Great Ormond Street Hospital Biomedical Research Centre, Great Ormond Street Institute of Child Health, University College London London UK; ^3^ MRC International Centre for Genomic Medicine in Neuromuscular Diseases London UK; ^4^ Queen Square Centre for Neuromuscular Diseases UCL Institute of Neurology London UK

## Abstract

**Objective:**

Characterise the diagnostic and prognostic value of muscle MRI patterns as biomarkers in a genetically heterogeneous nemaline myopathy (NM) patient cohort.

**Methods:**

Modified Mercuri scoring of lower limb MRI in genetically characterised NM patients referred to the highly specialised service for congenital myopathies at Great Ormond Street Hospital. Findings were compared to clinical data and MRI patterns derived from collated published data.

**Results:**

Twenty‐seven patients with MRI were identified (8 *NEB*‐NM, 13 *ACTA1*‐NM, 6 *TPM3*‐NM). *NEB*‐NM demonstrated sparing of the thigh. *ACTA*1‐NM demonstrated diffuse thigh involvement, notable in the vasti, sartorius and biceps‐femoris, with relative adductor and gracilis sparing. *TPM3*‐NM demonstrated diffuse thigh involvement notable in biceps‐femoris and adductor magnus with relative rectus femoris, adductor longus and gracilis sparing. In the lower leg, the soleus and tibialis anterior are notably involved in all three genotypes. *NEB*‐NM and *ACTA1*‐NM demonstrated relative gastrocnemii and tibialis posterior sparing, while *TPM3*‐NM showed significantly more tibialis posterior involvement (*P* =< 0.05). Comparison of involvement patterns with literature datasets highlighted preferential adductor and gracilis sparing in our *ACTA1*‐NM cohort, consistent tibialis posterior involvement in our *TPM3*‐NM cohort and a distinct MRI pattern from those derived from other NM genotypes and congenital myopathies. Greater tibialis anterior involvement correlated with foot drop (*P* = 0.02). Greater tibialis anterior and extensor hallucis longus involvement correlated with worse mobility (*P* =< 0.04).

**Interpretation:**

This is the widest NM MRI data set described to date; we describe distinct muscle involvement patterns for *NEB*‐NM, *ACTA1*‐NM and *TPM3*‐NM which may have utility as diagnostic and prognostic biomarkers and aid in genetic variant interpretation.

## Introduction

Nemaline myopathies (NM) are a subgroup of congenital myopathies, histopathologically characterised by the presence of nemaline bodies, or rods, on modified Gömöri trichrome staining or electron microscopy of affected skeletal muscle.^1^ Clinically, NM are typically characterised by early onset hypotonia, skeletal muscle weakness and motor difficulties. Variants in at least 14 causative genes have been identified to date (*ACTA1*, *NEB*, *KLHL40*, *TPM2*, *TPM3*, *CFL2*, *TNNT1*, *LMOD3*, *KBTBD13*, *MYPN*, *TNNT3*, *RYR3*, *KLHL41*, *CAP2*, *MYO18B*) with *NEB* and *ACTA1* being the most common.^1^, ^2^, ^3^, ^4^

The diagnostic value of muscle MRI in identifying muscle involvement patterns has been recognised for several neuromuscular disorders including congenital myopathies.^5^, ^6^, ^7^, ^8^, ^9^, ^10^, ^11^, ^12^, ^13^ However, there are limited published data on muscle MRI involvement patterns in NM. The largest case series published to date is confined to 10 patients including 6 with *NEB*‐NM and 4 with *ACTA1*‐NM.^14^ Improved understanding of MRI patterns of muscle involvement may help to guide diagnostic genetic testing and aid interpretation of identified genetic variants of unknown significance. Furthermore, muscle MRI may also play a role as an inclusion criteria and/or outcome measure in future clinical trials.

The aim of this work is to describe muscle involvement patterns seen on T1‐weighted MRI of the lower limbs in patients with differing NM genotypes, and to evaluate their utility as potential diagnostic and prognostic biomarkers.

## Methods

Subjects were identified using an internal diagnostic database of patients seen by, or referred to, the highly specialised service for congenital myopathies and muscular dystrophies at Great Ormond Street Hospital, London, United Kingdom. Genotypic data were reviewed to ensure patients had a genetically confirmed diagnosis of NM, with variants considered as likely pathogenic or pathogenic according to American College of Medical Genetics (ACMG) criteria.^15^ Patients with available lower limb T1‐weighted MRI were selected. MRIs were reviewed using the hospitals' digital PACS imaging system or, where only hard copies of historic MRIs were available, using a radiology light box. A single assessor (LP), trained in muscle MRI analysis, performed qualitative modified Mercuri scoring (MMS) (Table 1) of muscles of the pelvis/thigh (gluteal muscles, rectus femoris, vastus lateralis, vastus Intermedius, vastus medialis, sartorius, gracilis, biceps femoris, semitendinosus, semimembranosus, adductor magnus and adductor longus) and lower leg (tibialis anterior, extensor digitorum longus, peroneal muscles, tibialis posterior, soleus, lateral and medial gastrocnemii). Gluteal, peroneal and the long and short heads of the biceps femoris muscles were considered as a singular muscle entity and scored accordingly. Five (19%) MRIs underwent scoring by a second blinded assessor experienced in muscle MRI analysis (SS) and inter‐rater reliability was assessed using linearly weighted Cohen's Kappa. Results were analysed according to genotype, including mean modified Mercuri score (mMMS) and percentage occurrence of each MMS for each muscle. Muscles with MMS ≥ 2 were considered as notably involved, while muscles with scores of <2 were considered as spared. MMS for each muscle were plotted against disease duration for each genotype to assess for correlation.

**Table 1:** Modified Mercuri score (MMS) description.

Modified Mercuri score	Definition
0	Normal appearance
1	Early fat infiltration, scattered areas of T1 high signal
2	Numerous discrete areas of T1 high signal with beginning confluence <30% of the volume
3	Fat infiltration 30–60% of volume
4	Fat infiltration >60% of the volume
5	End stage, no residual muscle tissue

Clinical data on motor ability (at time of MRI and at latest clinical review) and the distribution of muscle weakness were collected from clinical records and compared to muscle Mercuri scores to assess for correlations. Motor abilities were stratified into 3 groups: ambulant (able to run, walk unlimited distances or walk >20 min duration), limited ambulation (walk independently <20 min, <100 m or require orthotics to ambulate) and non‐ambulant. The presence/absence of foot drop was also noted.

We performed statistical analysis of the data from our patient cohort. The Fisher exact test was applied to the contingency table comparing individual muscle MMS between genotypes. Due to small sample sizes, a Bonferroni correction was not used, and instead a universal significance threshold of 0.05 was considered. A Boolean PubMed literature search was performed using the following terms: (MRI/Magnetic resonance) AND (Nemaline, *NEB*, nebulin, *ACTA1*, alpha actin, *TPM2*, *TPM3*, tropomyosin, *TNNT1*, Troponin, *CFL2*, Cofilin 2, *KBTBD13*, *KLHL40*, *KLHL41*, Kelch, *LMOD3*, Leiomodin, *MYPN*, myopalladin, *TNNT3*, *MYO18B*, *RYR3*, *CAP2*). This search was aimed to identify all publications containing data on the pattern of muscle involvement on lower limb MRI from patients with NM. All retrieved publications were reviewed, and the described muscle involvement patterns recorded in a dedicated database. As complete Mercuri scoring (MS) or equivalent qualitative scoring was not consistently applied within the source literature, we devised a standardised scoring system to record these data. Within the source literature, muscles which were recorded as notably involved were given a score of +1; muscle recorded as notably spared were scored as −1, and muscles not mentioned as notably involved or spared were given a score of 0 (note, this does not preclude their involvement) (Table S4). Cumulative involvement score(s) (CIS) for each muscle were calculated, with higher positive scores indicating a higher rate of notable involvement, and negative scores indicating a higher rate of sparing/relative sparing, in the source literature. Results of the analysis of the internal patients' cohort were then compared to literature analysis. Statistical comparison between our study cohort and the literature data was not performed, owing to the heterogeneous nature of the literature data and the consequent lack of equivalence between the two data sets.

## Results

### Study cohort

The study cohort included 27 patients with a definitive genetic diagnosis of NM and available lower limb T1‐weighted muscle MRI: 13 with *ACTA1*‐NM, 8 with *NEB*‐NM, 6 *TPM3*‐NM (Table S1). Median ages at the time of MRI for each genotype were 11.5 years for *NEB*‐NM (IQR: 8.5, range: 6–16); 10 years for *ACTA1*‐NM (IQR: 11, range: 2–48); 23.5 years for *TPM3*‐NM (IQR: 39, range: 11–60). Median time from symptom onset to MRI for each genotype was 9.25 years for *NEB*‐NM (IQR: 8.29, range: 4–15.5); 9 years for *ACTA1*‐NM (IQR: 4.34, range: 2–46); 23.3 years for *TPM3*‐NM (IQR: 31, range: 11–50). MRI scoring inter‐rater reliability was 0.93 (95% CI: 0.89–0.98; *P* =< 0.001).

The range of motor abilities at time of MRI included fully ambulant (14/27), limited ambulation (8/27) and non‐ambulant (5/27) patients. The clinical pattern of muscle weakness included proximal (1/27), distal (1/27), equal proximal and distal (9/27), proximal>distal (8/27) and distal>proximal (8/27). Foot drop was present in 9/27 patients (Table S1).

**Figure 1:**
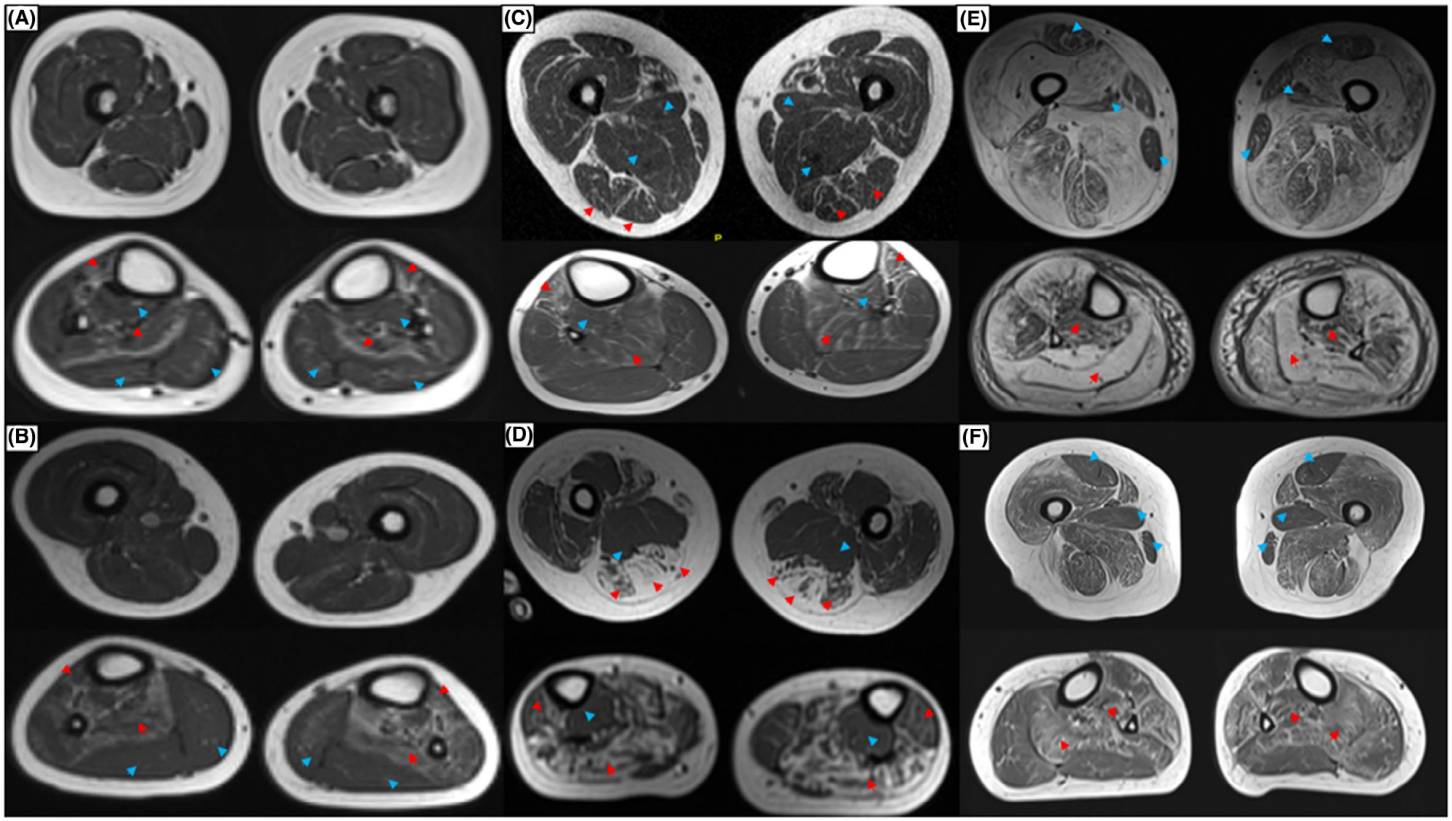
T1‐weighted lower limb MRI in NM patients from our cohort. Panels A (patient 2, 6 years old) and B (patient 1, 6 years old): *NEB*‐NM patients. Note the comparative sparing of the thigh muscles and the involvement of the tibialis anterior and soleus (red arrowheads) in both. Note the comparative sparing of the gastrocnemii in both and the tibialis posterior in patient 1 (blue arrow heads). Panels C (patient 11, 9 years old) and D (patient 15, 24 years old): *ACTA1*‐NM patients. Note the involvement of the hamstring muscles in the thigh (red arrow heads) and comparative sparing of the adductors (blue arrowheads). In the lower leg, note the involvement of the tibialis anterior and soleus (red arrowheads). Also, note comparative sparing of the tibialis posterior muscle (blue arrowheads). Panels E (patient 22, 60 years old) and F (patient 27, 51 years old): *TPM3*‐NM patients. Note the diffuse muscle involvement in the thigh with comparative sparing of the rectus femoris, gracilis and adductor longus muscles in both (blue arrowheads), and the involvement of the tibialis posterior and soleus muscles in the lower leg (red arrowheads).

**Figure 2:**
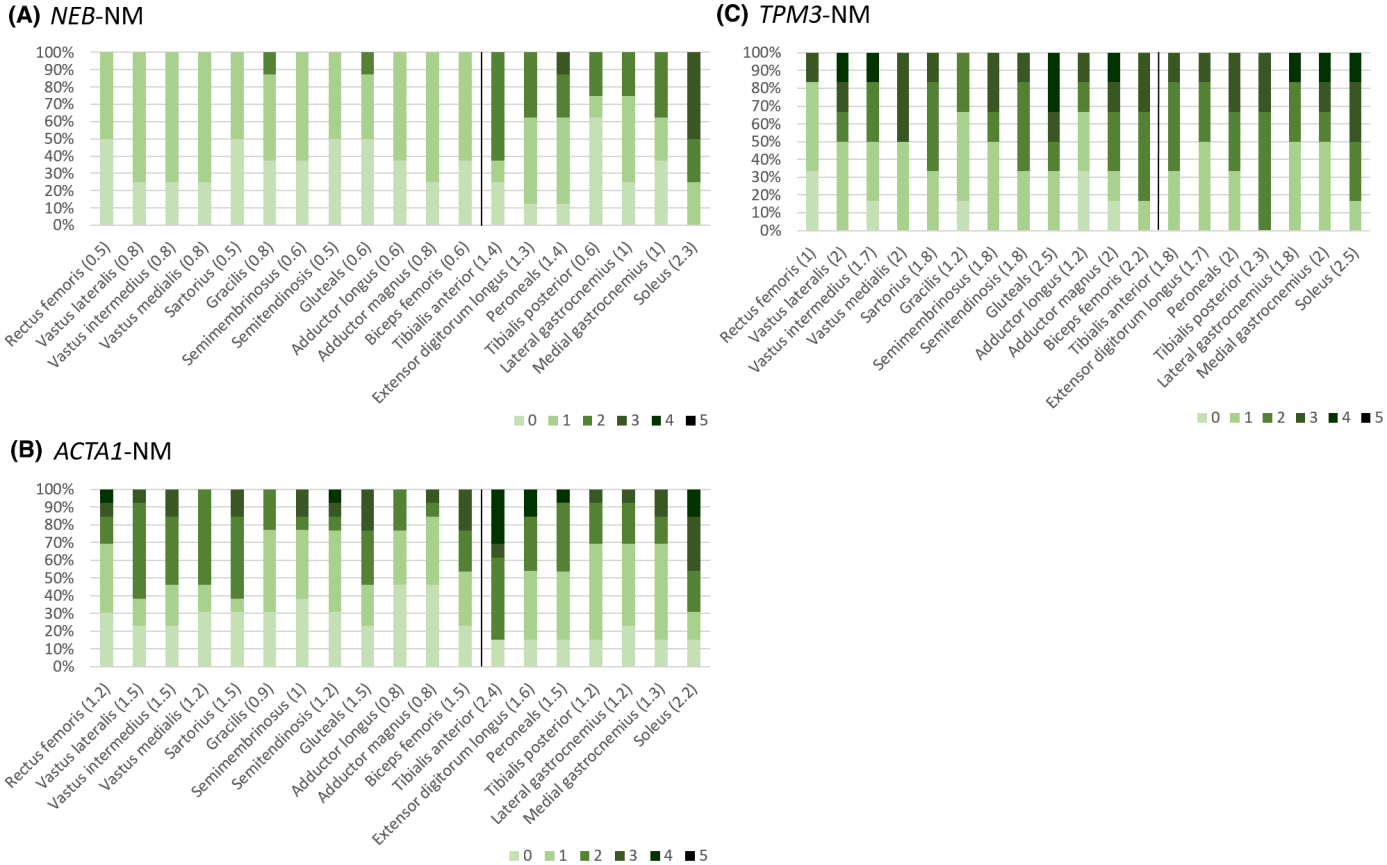
Modified Mercuri scores from our cohort by genotype. Frequency of modified Mercuri scores for individual muscles represented as a percentage of the total for each NM genotype for present cohort of NM patients. Panel A: *NEB*‐NM; Panel B: *ACTA1*‐NM, Panel C: *TPM3*‐NM. Modified Mercuri scores 0–5 are indicated with green bars (see scale), mean modified Mercuri scores for each muscle are depicted in brackets () along the *x* axis. Vertical black line delineates thigh from lower leg muscles.

**Figure 3:**
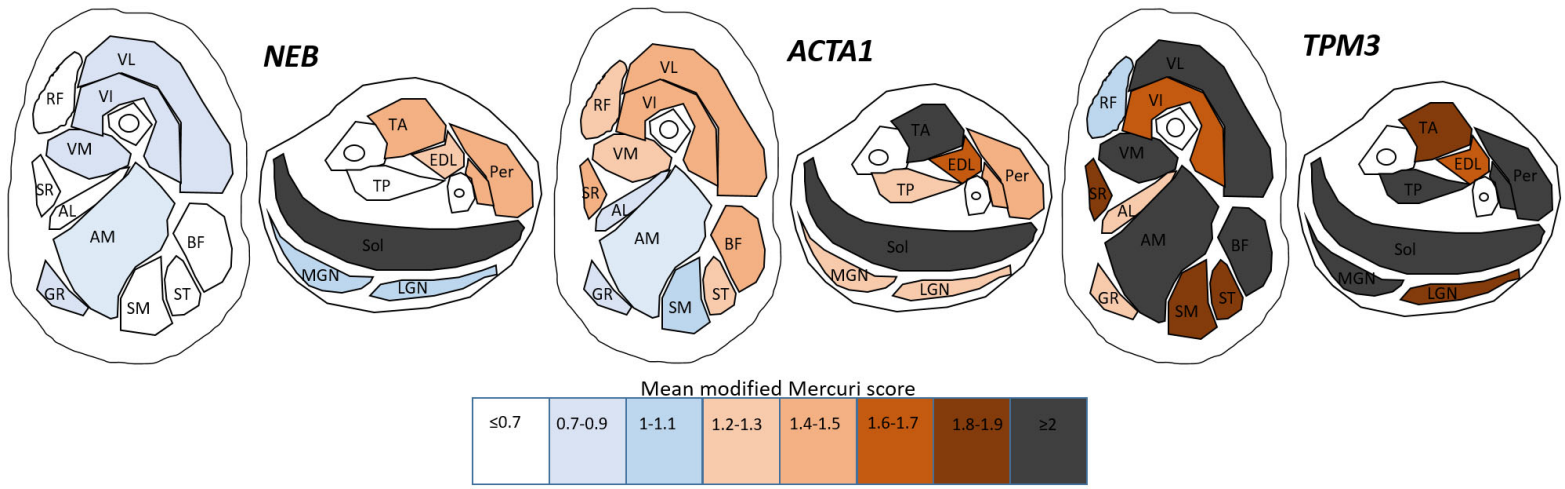
Graphical representation of mean modified Mercuri scores from our cohort by genotype. Cross‐sectional view through the thigh and lower leg for NEB‐, *ACTA1*‐ and *TPM3*‐NM patients from our current cohort. Muscle colours reflect mMMS (see colour scale for corresponding mMMS range). Rectus femoris (RF), vastus lateralis (VL), vastus intermedius (VI), vastus medialis (VM), sartorius (SR), adductor longus (AL), adductor magnus (AM), gracilis (GR), semimembranosus (SM), semitendinosus (ST), biceps femoris (BF), tibialis anterior (TA), tibialis posterior (TP), peroneal (Per), soleus (Sol), medial gastrocnemius (MGN), lateral gastrocnemius (LGN).

*NEB*‐NM patients presented a distal involvement pattern with universal sparing/minimal involvement of all thigh muscles (mMMS: 0.5–0.8). In the lower leg, mMMS were greatest for the soleus (mMMS: 2) and tibialis anterior (mMMS: 1.4) muscles. The soleus was notably involved in 5/8 (62.5%) of patients. The tibialis posterior (mMMS: 0.6) and gastrocnemii (mMMS: 1) were comparatively spared in >60% of patients. While the peroneal muscles demonstrated one of the highest mMMS (1.4), they were also comparatively spared in >60% of patients (Figs. 1, 2, 3 and Table S2).

*ACTA1*‐NM patients demonstrated frequent diffuse involvement of the thigh muscles (mMMS: 0.8–1.5). Within the pelvis/thigh, mMMS were greatest for the gluteal, vastus lateralis, vastus intermedius, sartorius and biceps femoris (mMMS: 1.5) muscles, with frequent relative sparing of the adductor (mMMS: 0.8) and gracilis (mMMS: 0.9) muscles. The adductor magnus was spared in 11/13 (85%), and adductor longus and gracilis muscles were spared in 10/13 (77%) patients. The anterior compartment in the lower leg (excluding the tibialis posterior) and soleus muscles were the most frequently involved, with lesser involvement of the gastrocnemii and tibialis posterior, muscles. The tibialis anterior (mMMS: 2.4) and soleus (mMMS: 2.2) muscles demonstrated the greatest mMMS and were notably involved in 11/13 (85%) and 9/13 (69%) patients, respectively. In contrast, the gastrocnemii (mMMS: 1.2–1.3) and tibialis posterior (mMMS: 1.2) muscles were spared/minimally involved in 9/13 (69%) patients (Figs. 1, 2, 3 and Table S2).

*TPM3*‐NM patients demonstrate frequent diffuse involvement of the thigh muscles, with greatest mMMS for the gluteal (mMMS: 2.5), biceps femoris (mMMS: 2.2), adductor magnus, vastus lateralis and vastus medialis muscles (mMMS: 2). The biceps femoris muscle was notably involved in 5/6 (83%) patients. In contrast, within the thigh, rectus femoris (mMMS: 1), gracilis and adductor longus (mMMS: 1.2) muscles demonstrated the lowest mMMS, with rectus femoris recorded as spared/minimally involved in 5/6 (83%) patients. Frequent notable involvement of the adductor magnus muscle was observed in 4/6 (67%) patients. Within the lower leg, the soleus (mMMS: 2.5) was one of the most involved muscles and was recorded as notably involved in 5/6 (83%) patients. The tibialis posterior muscle (mMMS: 2.3) was notably involved in 6/6 (100%) *TPM3*‐NM patients.

We performed three‐way and pairwise comparisons of muscle involvement for the genotypes within our patient cohort (*ACTA1*‐NM, *NEB*‐NM, *TPM3*‐NM). The Fisher exact test identified statistically significant differences in the severity of involvement between genotypes for the vastus medialis (*P* = 0.001), tibialis posterior (*P* = 0.008), vastus lateralis (0.014) and sartorius (0.026) muscles. In the thigh, the sartorius was significantly less involved in *NEB*‐NM compared to *ACTA1*‐NM (*P* = 0.024) and *TPM3*‐NM (*P* = 0.024). The vastus medialis and vastus lateralis were significantly more involved in *ACTA1*‐NM compared to *NEB*‐NM (*P* = 0.011 and *P* = 0.015 respectively). There was also significantly more involvement of the vastus medialis muscle in *TPM3*‐NM compared to *ACTA1*‐NM (*P* = 0.002). There was significantly more involvement of the biceps femoris and semitendinosus muscles in *TPM3*‐NM compared to *NEB*‐NM (*P* = 0.011 and *P* = 0.024 respectively). In the lower leg, the tibialis posterior muscle was significantly more involved in *TPM3*‐NM compared to both *NEB*‐NM (*P* = 0.023) and *ACTA1*‐NM (0.041). Other pairwise comparisons of muscle involvement did not reach statistical significance (Table S3).

We looked at associations between MRI pattern, distribution of clinical weakness and motor abilities, and correlated this to the genetics findings. The pattern of muscle involvement on MRI was largely consistent with patterns of clinical weakness. The two patients with isolated distal (patient 10) or proximal (patient 13) weakness demonstrated concordant distribution findings on muscle MRI. Analysis of the mean MMS for all thigh and lower leg muscles for other patterns of clinical weakness consistently demonstrated a higher mean score of involvement for the lower leg. The mean MMS for the lower leg and difference between thigh and lower leg mean MMS were marginally greater for patients with a distal>proximal pattern of weakness compared to those with a proximal>distal weakness pattern (4.63 vs. 4.42, and 1.28 vs. 1.2). The presence of foot drop was significantly associated with higher MMS in the tibialis anterior (*P* = 0.02) and all patients with clinical foot drop had an MMS ≥ 2 in this muscle (9/9).

There was a statistically significant correlation between the degree of involvement of the tibialis anterior and extensor digitorum longus muscles and motor function, with higher MMS in these two muscles corresponding to worse mobility (*P* = 0.04 and *P* = 0.02 respectively). All patients that were not ambulant at the time of MRI had an MMS ≥ 2 in the tibialis anterior muscle (13/13), and similarly all patients with an MMS < 2 in this muscle were ambulant at the time of MRI (7/7). There was no significant correlation between individual thigh muscle involvement and mobility. Half (7/14) of patients who were ambulant at the time of MRI had an MMS ≥ 2 in the tibialis anterior. Of those patients who were ambulant at the time of MRI and had an MMS < 2 in the tibialis anterior, 6/7 (85%) remained ambulant at follow‐up (median age at MRI 12 years, median time between MRI and follow‐up 3.5 years). This is in contrast to what was observed in patients who were still ambulant at time of MRI and had a tibialis anterior MMS ≥ 2, in whom 6/7 (85%) subsequently had limited ambulation (5/6) or lost ambulation (1/6) on follow‐up (median age at MRI 25.7 years; median time between MRI and follow‐up 6.5 years). Owing to the numerous confounding factors associated with these observations, including age at MRI and length of follow‐up, further statistical analysis was not performed.

### Literature review

**Figure 4:**
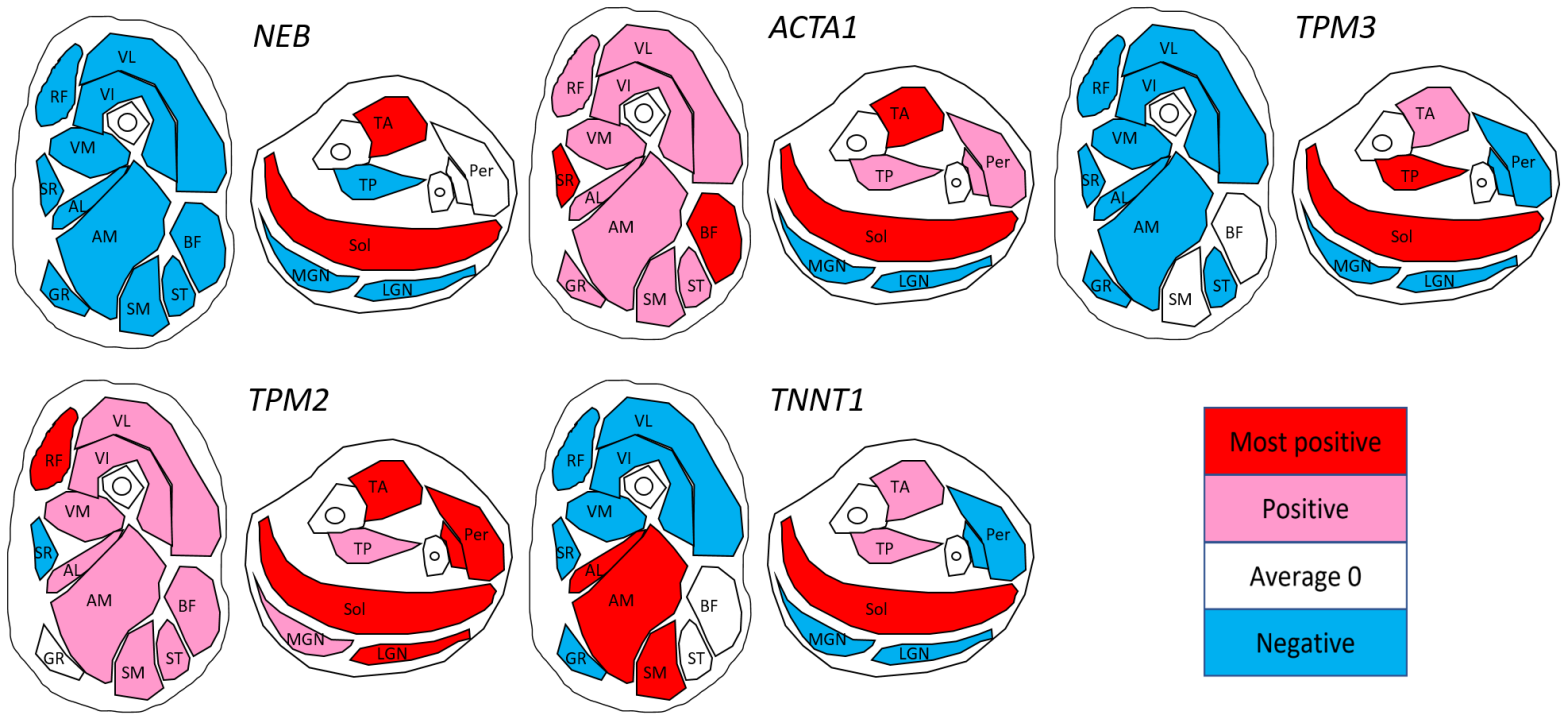
Graphical representation of pattern of muscle involvement by genotype from collated literature data. Figures represent cross‐sectional view through the thigh and lower leg for each genotype. Muscle in blue represents negative CIS, white represents a CIS of 0, pink represents a positive CIS and red represents those muscles with the most positive CIS for that genotype. Full methodology for CIS scoring and raw data is outlined in Table S4.

Review of the literature yielded 30 publications containing data on patterns of muscle involvement on lower limb MRI for genetically characterised NM patients.^14^, ^16^, ^17^, ^18^, ^19^, ^20^, ^21^, ^22^, ^23^, ^24^, ^25^, ^26^, ^27^, ^28^, ^29^, ^30^, ^31^, ^32^, ^33^, ^34^, ^35^, ^36^, ^37^, ^38^, ^39^, ^40^, ^41^, ^42^, ^43^, ^44^ These included MRI data for 64 genotyped patients: 16 *NEB*‐NM, 14 *ACTA1*‐NM, 19 *TPM2*‐NM, 5 *TTNT1*‐NM, 6 *TPM3*‐NM, 2 *KBTBD13*‐NM, 1 *LMOD3*‐NM, 1 *KLHL40*‐NM. Figure 4 graphically represents the findings of this analysis and the raw data are displayed in Table S4. There was no significant difference in age at MRI between our patients and those from the literature, including when assessing genotype specific cohorts (data not shown).

## Discussion

In this work, we present the widest data set of lower limb muscle MRI patterns for NM patients published to date and offer a detailed cumulative analysis of and comparison with muscle MRI literature data. Analysis of our patients' cohort highlights distinct muscle involvement patterns for *NEB*‐NM, *ACTA1*‐NM and *TPM3*‐NM. Overall, when comparing MRI findings in these three genotypes within our cohort, we highlight notable involvement of the tibialis anterior and soleus muscles in all (Figs. 2A–C and 3). Comparative sparing of the thigh musculature was more often seen in *NEB*‐NM, and while not reaching clinical significance, possibly caused by the small sample size studied, this appeared to be a consistent finding. Although both *ACTA1*‐NM and *TPM3*‐NM frequently showed diffuse thigh involvement, the vastus medialis was significantly more involved in *TPM3*‐NM (*P* = 0.002), suggesting that involvement of this muscle may help to differentiate between these two genotypes. Similarly, our cohort analysis also suggests notable involvement of the adductor magnus within the thigh as evocative of *TPM3*‐NM (as notably involved in 60% of patients) rather than *ACTA1*‐NM (spared in 85%). However, this finding failed to reach statistical significance (*P* = 0.176). Within the lower leg, our data highlight a striking difference in tibialis posterior involvement between the three genotypes, with this muscle always involved in *TPM3*‐NM and comparatively spared in the other two (Figs. 1, 2 and 3). This finding suggests that involvement of the tibialis posterior muscle may help to differentiate between these genotypes and serve as a potential diagnostic biomarker for *TPM3*‐NM. We did not find any correlation between degree of muscle fat infiltration and disease duration in the three genotypes (data not shown).

We found a significant association between greater tibialis anterior involvement on MRI and foot drop, and greater tibialis anterior and extensor digitorum longus involvement and worse mobility. This finding may suggest that impairment of ankle dorsiflexion, even to a degree less than complete foot drop, may be an early contributing cause of impaired ambulatory capability in NM patients. Our data highlight a possible association between greater tibialis anterior involvement (MMS ≥ 2) and subsequent ambulatory deterioration in patients who were ambulant at the time of MRI. However, this observation may reflect the older age and longer follow‐up period associated with this group.

The muscle involvement patterns observed in *NEB*‐NM and *ACTA1*‐NM in our patient cohort were largely consistent with the results we obtained from the analysis of collated published data for these genotypes. In particular, for *NEB*‐NM, analysis of both data sets highlighted sparing of the thigh muscles, gastrocnemii and tibialis posterior, in association with frequent notable involvement of the soleus and tibialis anterior muscles (Figs. 2A, 3, 4). For *ACTA1*‐NM, both data analyses highlight diffuse involvement of the thigh musculature with notable involvement of the biceps femoris and sartorius. Similarly, notable involvement of the tibialis anterior and soleus muscles and comparative sparing of the gastrocnemii was noted in both data sets. However, in contrast to collated published data, our cohort highlights comparative sparing of the adductor and gracilis muscles.

The literature analysis of *TPM3*‐NM yielded numerous similarities with the data from our patient cohort, including sparing of the rectus femoris and gracilis muscles in the thigh, and notable involvement of the soleus, tibialis posterior and tibialis anterior muscles in the lower leg. Of note, involvement of the tibialis posterior muscle was only commented upon in two of the six available published *TPM3*‐NM patient reports. Of the remaining 4 patients, MRI (single slice) images of the lower leg were only available for two, and suggest tibialis posterior involvement in one and possibly in the other, although this was not commented in the original publication.^37^ In contrast to the findings from our cohort, the literature analysis suggested more diffuse sparing of the thigh musculature. However, the biceps femoris was one of only two muscles not to demonstrate a negative CIS and could be seen to be in keeping with higher level of involvement of this muscle demonstrated in our patient cohort. The literature review also highlighted sparing of the adductor muscles which is in keeping with the sparing of the adductor longus, but in contrast to frequent involvement of the adductor magnus seen within our cohort. It should be noted that the available literature did not distinguish between the adductor longus and adductor magnus muscles when reporting MRI findings.

The muscle involvement patterns generated from our patient cohort appear distinct from those generated from the literature of other NM genotypes (Figs. 3 and 4) and those commonly associated with other congenital myopathies and muscular dystrophies.^7^, ^45^ One exception is the isolated distal lower limb involvement, predominantly of the anterior compartment, seen in around 12% of patients with Laing distal myopathy due to pathogenic *MYH7* gene variants, which may mimic the imaging findings seen in *NEB*‐NM.^46^ However, Laing myopathy patients may also demonstrate an inverted or true collagen VI sign which is absent in *NEB*‐NM and was not seen in any of our patients.^46^ We also noted a similarity in the pattern of muscle involvement between *RYR1*‐related congenital myopathy and the *TPM3*‐NM group presented here. In particular, we demonstrated relative sparing of the rectus femoris, comparative lesser involvement of the adductor longus compared to the adductor magnus, involvement of the sartorius, comparative sparing of the gracilis and notable involvement of the soleus in the lower leg; findings that are similar to those observed in *RYR1* congenital myopathy (Fig. 3).^10^, ^47^ Conversely, MRI features helping differentiating between these two genotypes include the relative higher degree of involvement of the biceps femoris, tibialis anterior and tibialis posterior muscles in the *TPM3*‐NM cohort versus the typical sparing of these muscles in *RYR1* myopathy.^47^

The pattern of muscle involvement seen in *NEB*‐NM was reasonably homogeneous both within our study cohort and within the literature (Figs. 3 and 4, and Tables S2 and S4). Conversely, we noted a degree of heterogeneity in MRI patterns in *ACTA1*‐NM and *TPM3*‐NM cohorts. In particular, we noted that some *TPM3*‐NM patients exhibited sparing of the adductors (patient 25) and notable involvement of the rectus femoris (patient 22). In the *ACTA1*‐NM cohort, patient 10 had the most atypical MRI pattern, with sparing of the proximal upper and proximal lower limbs' muscles, but with notable distal involvement (Table S2). In keeping with this MRI pattern, the patient presented at 18 months of age with a clinically distal phenotype with foot drop and subgravity finger power. Such distal presentation is atypical of *ACTA1*‐NM, and in the absence of genetic diagnosis, may be considered more in keeping with *NEB*‐NM. This MRI variability highlights that while the findings of this study may be of utility in differentiating between NM genotypes, patterns of muscle involvement are not universal nor pathognomonic.

The differences observed between our cohort and reported literature may be due to a number of factors, including the relatively small number of reported patients with MRI, the limited published MRI data, variation in methods of MRI analysis employed, the depiction of MRI data within the source literature and the limitations associated with applying a uniform scoring system (CIS) to heterogeneous published data.

Despite some intra‐genotypic heterogeneity, the imaging pattern of NM and other congenital myopathies can play an integral role in the integrated multidisciplinary, clinico‐genomic diagnostic approach to patients. Given the size and complexity of many neuromuscular genes, and the increasing number of variants of uncertain significance (VUS) identified through next‐generation sequencing, muscle MRI provides further depth to the deep phenotypisation of genetically undiagnosed patients. In turn, identification of gene‐specific imaging patterns or ‘imaging fingerprints’ may assist interpretation of VUS.^15^

There are inherent limitations of qualitative MRI grading scales, including MMS, including in particular the subjective nature of these scales, the need to have consistency training sessions to improve inter‐rater variability, and the fact that these are limited to (typically) a maximum 4–6 grades differentiable by the human eye. Quantitative assessment of muscle fat fraction has been made possible using Dixon sequences which have a high reproducibility and reliability with low inter‐ and intra‐observer difference.^48^, ^49^ Quantitative MRI analysis in research has indeed been demonstrated to be of prognostic value for several NMD.^12^, ^50^, ^51^, ^52^ Owing to the rarity of NM and the paucity of available muscle imaging, the current study was limited to clinical T1‐weighted MRI images. However, prospective serial quantitative muscle MRI will be of value in confirming the role of MRI as a diagnostic and prognostic biomarker in NM.

In conclusion, we define distinct patterns of muscle involvement in NM that may be of utility as diagnostic biomarkers. Further studies on larger, possibly international case series are warranted to confirm the validity of these findings, further evaluate genotype–phenotype correlations and investigate the possible role of muscle MRI as prognostic biomarker and outcome measure for these rare conditions.

## Author contributions

All authors have contributed to the conception and design of the study. JM, SS and LS assisted in editing of the manuscript. LP, GS, GB, FM and AS assisted in data analysis and editing of the manuscript.

## Conflict of interest

None to declare.

## Supporting information


**Table S1.** Patient demographic and genotypic information for our patient cohort. M (male), F (female), AFOs (ankle foot orthoses), N/A (data not available).

**Table S2.** Raw modified Mercuri scores from our cohort. Raw MMS (0–5) for our patient cohort are indicated for each patient. White boxes denote sparing of the muscle (MMS 0). Pink to deep red boxes denote increasing MMS with darker shades representing higher scores (MMS 1–5). Mean MMS for each muscle for each genotype are highlighted in green. Rectus femoris (RF), vastus lateralis (VL), vastus intermedius (VI), vastus medialis (VM), sartorius (SR), adductor longus (AL), adductor magnus (AM), gracilis (GR), semimembranosus (SM), semitendinosus (ST), biceps femoris (BF), tibialis anterior (TA), tibialis posterior (TP), peroneal (Per), extensor digitorum longus (EDL), soleus (SOL), medial gastrocnemius (MGN), lateral gastrocnemius (LGN).

**Table S3.** Fisher exact test *P*‐value scores for three‐way and pairwise comparisons. Pink‐highlighted boxes represent statistically significant values (*P* =< 0.05).

**Table S4.** Involvement score data from source literature by genotype. Where full Mercuri scoring (MS) or equivalent qualitative muscle MRI scoring was available within the source literature, all muscles recorded as having an MS ≥ 2 or equivalent were attributed a score of +1, unless they are commented upon as being notably comparatively spared, in which case they were attributed a score of −1. Muscles with MS of 1 were attributed a score of 0, unless they are remarked upon as notably involved or spared compared to other muscles in which case they were scored as +1 or −1 accordingly. Muscles with an MS of 0 were given a score of −1. Where full MS or equivalent was not available, muscles that have been notably mentioned as involved within the source literature were attributed a score of +1; those mentioned as spared or relatively spared were attributed a score of −1; those muscles not explicitly specified as particularly involved or spared were attributed a score of 0 (note this does not preclude their involvement). Where the source literature references that findings are compatible/classical of a specified muscle involvement pattern—those muscles considered to be involved or spared as part of this referenced pattern were attributed scores of +1 or −1 accordingly. Where the source literature states muscle groups (e.g. quadriceps, hamstring muscles) were diffusely involved/spared, the muscles constituting these groups were attributed a score of +1/−1 accordingly. Red boxes represent scores of +1, blue boxes represent scores of −1, white boxes represent scores of 0. Green rows represent cumulative scores for each muscle according to genotype. Rectus femoris (RF), vastus lateralis (VL), vastus intermedius (VI), vastus medialis (VM), sartorius (SR), adductors (Add), gracilis (GR), semimembranosus (SM), semitendinosus (ST), biceps femoris (BF), tibialis anterior (TA), tibialis posterior (TP), extensor digitorum longus (EDL), peroneal (PER), soleus (SOL), medial gastrocnemius (MGN), lateral gastrocnemius (LGN). Green rows highlight cumulative scores for each genotype.
