# Supplementary material for: Muscle magnetic resonance imaging involvement patterns in nemaline myopathies
Source: Ann Clin Transl Neurol. 2023 Jun 2;10(7):1219–29. doi: 10.1002/acn3.51816 (PMC10351659; doi:10.1002/acn3.51816)
Supplement: Supplementary file 1 — Table S1. Patient demographic and genotypic information for our patient cohort. M (male), F (female), AFOs (ankle foot orthoses), N/A (data not available). [file ACN3-10-1219-s004.docx]

**Supplemental table S1: Patient demographic and genotypic information for our patient cohort**

| Patient | Sex | Gene | Variant 1 | Variant 2 | Age at first concerns | Age at MRI (years) | Pattern of weakness In lower limbs | Foot drop | Mobility at time of MRI | Age at last assessment | Mobility at last assessment |
| --- | --- | --- | --- | --- | --- | --- | --- | --- | --- | --- | --- |
| 1 | M | *NEB* | Exon 55 deletion | c.24267_24270dup (p.Val8091fs) | 18 months | 6 | distal>proximal | Yes | Walk for 2 miles | 23 | Walk 20 meters |
| 2 | M | *NEB* | c.24454C>T p.(Arg8152Ter) | c.24454C>T p.(Arg8152Ter) | 9 months | 6 | distal>proximal | Yes | Walk 100 meters | 18 | walk in AFOs |
| 3 | F | *NEB* | c.3879+1G>A | c.3879+1G>A | 9 months | 14 | Proximal=distal | No | Walk for 30minutes | 18 | Walk for 30 minutes |
| 4 | F | *NEB* | c.2415+1G>A | c.2415+1G>A | <6 months | 6 | Proximal>distal | No | Slow run | 9 | Slow run |
| 5 | F | *NEB* | c.78+1G>A | c.24372_24375del p.(Arg8125fs) | 5 years | 9 | Proximal>distal | No | Slow run | 9 | Slow run |
| 6 | M | *NEB* | c.2415+1G>A | c.2415+1G>A | 2 years | 15 | Proximal>distal | No | Not available | 24 | Wheelchair dependant |
| 7 | F | *NEB* | c.24129_24133dup p.(Tyr8045fs) | c.6702+1G>T | 6 months | 16 | proximal=distal | Yes | Walk with AFOs indoors | 19 | Wheelchair dependant |
| 8 | M | *NEB* | c.3255+1G>T | c.3255+1G>T | 11 months | 14 | proximal=distal | No | Walk independently for 15 minutes | 23 | Walk 20 meters |
| 9 | M | *ACTA1* | c.16G>A p.(Glu6Lys) | - | <28 days | 10 | proximal=distal | No | Walk independently for 20 minutes | 15 | Walk independently (20 minutes) |
| 10 | M | *ACTA1* | c.280A>G p.(Asn94Asp) | - | 18 months | 5 | Distal | Yes | Walk unlimited in AFOs | 9 | Walk 3 miles in AFOs |
| 11 | M | *ACTA1* | c.715G>A p.(Glu239Lys) | - | <28 days | 9 | distal>proximal | Yes | Wheelchair dependant | 9 | Wheelchair dependant |
| 12 | F | *ACTA1* | c.983-985del (p.Lys328del) | - | 2 years | 12 | distal>proximal | Yes | Walk for 1 hour in AFOs | 12 | Walk for 1 hour (AFOs) |
| 13 | F | *ACTA1* | c.287T>C p.(Leu96Pro) | - | <28 days | 14 | proximal | No | Walk independently for 30 minutes | 15 | Walk independently for 30 minutes |
| 14 | M | *ACTA1* | c.599A>G p.(Tyr200Cys) | - | <28 days | 9 | Proximal>distal | No | Walk 200 meters in AFOs | 17 | Wheelchair dependant |
| 15 | F | *ACTA1* | c.782A>T p.(Glu261Val) | - | N/A | 24 | Proximal=distal | Yes | Walk unlimited | 28 | Walk independently with AFOs (unlimited) |
| 16 | F | *ACTA1* | c.16G>A (p.Glu6Lys) | - | 8 months | 12 | Proximal>distal | No | Walk for 2 hours | 17 | Wheelchair dependant |
| 17 | F | *ACTA1* | C.400A>G p.(Met134Val) | - | 3 years | 40 | Proximal=distal | No | Walk independently 1 mile | 47 | Walk independently (50 yards) |
| 18 | F | *ACTA1* | c.803T>C p.(Phe268Ser) | - | <28 days | 9 | Proximal=distal | No | Wheelchair dependant | 9 | Wheelchair dependant |
| 19 | F | *ACTA1* | c.803T>C p.(Phe268Ser) | - | <28 days | 7 | Proximal=distal | No | Wheelchair dependant | 7 | Wheelchair dependant |
| 20 | F | *ACTA1* | c.616G>A p.(Ala206Thr) | - | 2 years | 48 | Distal>proximal | Yes | Walk independently with AFOS (<20 minutes) | 50 | Walk independently with AFOS (<20 minutes) |
| 21 | M | *ACTA1* | c.280_282 del p.(Asn94Del) | - | <28 days | 2 | Proximal>distal | N/A | Unable to sit | 3 | Unable sit |
| 22 | M | *TPM3* | c.8A>G p.(Glu3Gly) | - | 17 years | 60 | Distal>proximal | Yes | Walk independently | 69 | Walk independently with AFOs (<20 minutes) |
| 23 | F | *TPM3* | c.502C>T p.(Arg168Cys) | - | 4 months | 20 | Proximal>distal | No | Walk independently (unlimited) | 30 | Walk independently (unlimited) |
| 24 | F | *TPM3* | c.503G>A p.Arg168His) | - | <28 days | 12 | Proximal>distal | No | Walk independently (unlimited) | 18 | Walk independently 100 yards |
| 25 | F | *TPM3* | c.503G>A p.Arg168His) | - | 6 weeks | 27 | Distal>proximal | No | Walk independently (unlimited) | 31 | Walk independently (unlimited) |
| 26 | M | *TPM3* | Deletion of 2 exons | - | <28 days | 11 | Proximal>distal | No | Walk independently unlimited | 17 | Walk independently 50 meters |
| 27 | F | *TPM3* | c.502C>T p.(Arg168Cys) | - | 1 year | 51 | Proximal=distal | No | Walk independently (<20mins) | 50 | Walk independently (<20mins) |

Supplemental table S1 legend: M (male), F (Female) AFOs (ankle foot orthoses), N/A (data not available)
