# Supplementary material for: Muscle magnetic resonance imaging involvement patterns in nemaline myopathies
Source: Ann Clin Transl Neurol. 2023 Jun 2;10(7):1219–29. doi: 10.1002/acn3.51816 (PMC10351659; doi:10.1002/acn3.51816)
Supplement: Supplementary file 2 — Table S2. Raw modified Mercuri scores from our cohort. Raw MMS (0–5) for our patient cohort are indicated for each patient. White boxes denote sparing of the muscle (MMS 0). Pink to deep red boxes denote increasing MMS with darker shades representing higher scores (MMS 1–5). Mean MMS for each muscle for each genotype are highlighted in green. Rectus femoris (RF), vastus lateralis (VL), vastus intermedius (VI), vastus medialis (VM), sartorius (SR), adductor longus (AL), adductor magnus (AM), gracilis (GR), semimembranosus (SM), semitendinosus (ST), biceps femoris (BF), tibialis anterior (TA), tibialis posterior (TP), peroneal (Per), extensor digitorum longus (EDL), soleus (SOL), medial gastrocnemius (MGN), lateral gastrocnemius (LGN). [file ACN3-10-1219-s001.docx]

**Supplemental table S2: Raw modified Mercuri scores from our cohort.**

| **Patient** | **Gene** | **RF** | **VL** | **VM** | **VI** | **SR** | **ST** | **SM** | **GM** | **Gr** | **AL** | **AB** | **AM** | **BF** | **TA** | **EDL** | **TP** | **Per** | **LGN** | **MGN** | **SOL** |
| --- | --- | --- | --- | --- | --- | --- | --- | --- | --- | --- | --- | --- | --- | --- | --- | --- | --- | --- | --- | --- | --- |
| 1 | *NEB* | 0 | 1 | 1 | 1 | 0 | 1 | 1 | 0 | 0 | 1 | 1 | 1 | 1 | 2 | 1 | 2 | 3 | 1 | 2 | 3 |
| 2 | *NEB* | 1 | 1 | 1 | 1 | 0 | 1 | 1 | 1 | 1 | 1 | 1 | 1 | 1 | 2 | 2 | 0 | 1 | 2 | 2 | 3 |
| 3 | *NEB* | 1 | 1 | 1 | 1 | 1 | 1 | 1 | 1 | 1 | 1 | 1 | 1 | 1 | 0 | 1 | 0 | 1 | 1 | 1 | 1 |
| 4 | *NEB* | 0 | 1 | 0 | 0 | 1 | 0 | 0 |  | 2 | 0 | 0 | 1 | 0 | 1 | 1 | 0 | 1 | 0 | 0 | 2 |
| 5 | *NEB* | 0 | 0 | 0 | 0 | 0 | 0 | 0 | 0 | 0 | 0 | 0 | 0 | 0 | 0 | 0 | 0 | 0 | 0 | 0 | 2 |
| 6 | *NEB* | 0 | 0 | 1 | 1 | 1 | 1 | 1 | 0 | 1 | 1 | 1 | 1 | 1 | 2 | 2 | 0 | 2 | 1 | 0 | 1 |
| 7 | *NEB* | 1 | 1 | 1 | 1 | 1 | 0 | 1 | 2 | 1 | 1 | 1 | 1 | 1 | 2 | 1 | 1 | 1 | 1 | 1 | 3 |
| 8 | *NEB* | 1 | 1 | 1 | 1 | 0 | 0 | 0 | 1 | 0 | 0 | 0 | 0 | 0 | 2 | 2 | 2 | 2 | 2 | 2 | 3 |
| **Mean MMS** | ***NEB*** | **0.5** | **0.75** | **0.75** | **0.75** | **0.5** | **0.5** | **0.625** | **0.625** | **0.75** | **0.625** | **0.625** | **0.75** | **0.625** | **1.375** | **1.25** | **0.625** | **1.375** | **1** | **1** | **2.25** |
| 9 | *ACTA1* | 0 | 0 | 0 | 0 | 0 | 0 | 0 | 0 | 0 | 0 | 0 | 0 | 0 | 0 | 0 | 1 | 1 | 0 | 0 | 0 |
| 10 | *ACTA1* | 0 | 0 | 0 | 0 | 0 | 0 | 0 | 0 | 0 | 0 | 0 | 0 | 0 | 4 | 4 | 0 | 0 | 1 | 1 | 1 |
| 11 | *ACTA1* | 4 | 2 | 1 | 2 | 2 | 4 | 3 | 2 | 2 | 0 | 0 | 0 | 3 | 2 | 2 | 1 | 2 | 2 | 3 | 3 |
| 12 | *ACTA1* | 0 | 1 | 0 | 1 | 0 | 1 | 0 | 1 | 0 | 0 | 1 | 1 | 1 | 3 | 2 | 1 | 2 | 1 | 1 | 1 |
| 13 | *ACTA1* | 0 | 0 | 0 | 0 | 0 | 0 | 0 | 0 | 1 | 1 | 1 | 1 | 2 | 0 | 0 | 0 | 0 | 0 | 0 | 0 |
| 14 | *ACTA1* | 1 | 2 | 2 | 2 | 1 | 1 | 1 | 3 | 1 | 2 | 1 | 1 | 2 | 2 | 2 | 2 | 2 | 2 | 2 | 4 |
| 15 | *ACTA1* | 1 | 1 | 1 | 1 | 2 | 1 | 1 | 2 | 0 | 0 | 0 | 0 | 1 | 4 | 2 | 1 | 1 | 0 | 1 | 3 |
| 16 | *ACTA1* | 1 | 2 | 2 | 1 | 2 | 1 | 1 | 2 | 1 | 1 | 1 | 1 | 2 | 2 | 1 | 2 | 2 | 1 | 1 | 3 |
| 17 | *ACTA1* | 1 | 2 | 2 | 2 | 2 | 1 | 1 | 3 | 1 | 1 | 1 | 1 | 1 | 2 | 1 | 1 | 2 | 1 | 1 | 2 |
| 18 | *ACTA1* | 2 | 2 | 2 | 2 | 2 | 0 | 0 | 1 | 1 | 0 | 0 | 0 | 0 | 2 | 1 | 1 | 1 | 1 | 1 | 2 |
| 19 | *ACTA1* | 2 | 2 | 2 | 2 | 2 | 1 | 1 | 2 | 1 | 1 | 0 | 0 | 1 | 2 | 1 | 1 | 1 | 1 | 1 | 2 |
| 20 | *ACTA1* | 1 | 3 | 2 | 3 | 3 | 2 | 3 | 3 | 2 | 2 | 0 | 2 | 3 | 4 | 1 | 2 | 1 | 2 | 2 | 3 |
| 21 | *ACTA1* | 3 | 2 | 2 | 3 | 3 | 3 | 2 | 1 | 2 | 2 | 2 | 3 | 3 | 4 | 4 | 3 | 4 | 3 | 3 | 4 |
| **Mean MMS** | **ACTA1** | **1.23** | **1.46** | **1.23** | **1.46** | **1.46** | **1.2** | **1** | **1.54** | **0.92** | **0.77** | **0.54** | **0.77** | **1.46** | **2.38** | **1.62** | **1.23** | **1.46** | **1.15** | **1.31** | **2.15** |
| 22 | TPM3 | 3 | 4 | 3 | 4 | 3 | 3 | 3 | 4 | 2 | 3 | 4 | 4 | 3 | 3 | 3 | 3 | 3 | 4 | 4 | 4 |
| 23 | TPM3 | 0 | 1 | 1 | 1 | 2 | 2 | 2 | 2 | 2 | 2 | 2 | 2 | 2 | 1 | 1 | 2 | 1 | 1 | 1 | 1 |
| 24 | TPM3 | 1 | 1 | 1 | 1 | 1 | 1 | 1 | 1 | 1 | 1 | 1 | 1 | 1 | 2 | 2 | 2 | 2 | 1 | 1 | 2 |
| 25 | TPM3 | 1 | 1 | 1 | 0 | 2 | 1 | 1 | 1 | 0 | 0 | 0 | 0 | 2 | 2 | 1 | 2 | 1 | 1 | 1 | 2 |
| 26 | TPM3 | 1 | 2 | 3 | 2 | 1 | 2 | 1 | 3 | 1 | 1 | 2 | 3 | 2 | 1 | 1 | 3 | 2 | 2 | 3 | 3 |
| 27 | TPM3 | 0 | 3 | 3 | 2 | 2 | 2 | 3 | 4 | 1 | 0 | 2 | 2 | 3 | 2 | 2 | 2 | 3 | 2 | 2 | 3 |
| **Mean MMS** | **TPM3** | **1** | **2** | **2** | **1.7** | **1.8** | **1.8** | **1.8** | **2.5** | **1.2** | **1.2** | **1.8** | **2** | **2.2** | **1.8** | **1.7** | **2.3** | **2** | **1.8** | **2** | **2.5** |

Legend: Raw MMS (0-5) for our patient cohort are indicated for each patient. White boxes denote sparing of the muscle (MMS 0). Pink to deep red boxes denote increasing MMS with darker shades representing higher scores (MMS 1-5). Mean MMS for each muscle for each genotype are highlighted in green. Rectus femoris (RF), vastus lateralis (VL), vastus intermedius (VI), vastus medialis (VM), sartorius (Sart), adductor longus (AL), adductor magnus (AM), gracilis (GR), semimembranosus (SM), semitendinosus (ST), biceps femoris (BF), tibialis anterior (TA), tibialis posterior(TP), peroneal (Per), Extensor digitorum longus (EDL), soleus (SOL), medial gastrocnemius (MGaN), lateral gastrocnemius (LGaN).
