# Supplementary material for: Muscle magnetic resonance imaging involvement patterns in nemaline myopathies
Source: Ann Clin Transl Neurol. 2023 Jun 2;10(7):1219–29. doi: 10.1002/acn3.51816 (PMC10351659; doi:10.1002/acn3.51816)
Supplement: Supplementary file 3 — Table S3. Fisher exact test P‐value scores for three‐way and pairwise comparisons. Pink‐highlighted boxes represent statistically significant values (P =< 0.05). [file ACN3-10-1219-s002.docx]

**Supplemental table S3: Fisher exact test p-value scores for three-way and pairwise comparisons for our cohort**

| **Muscle** | **Three-way comparison** | **NEB/ACTA1** | ***ACTA1/TPM3*** | **NEB/TPM3** |
| --- | --- | --- | --- | --- |
| **Rectus femoris** | 0.935 | 0.78 | 1 | 0.767 |
| **Vastus lateralis** | **0.014** | **0.015** | 0.1 | 0.203 |
| **Vastus intermedius** | 0.147 | 0.064 | 0.703 | 0.189 |
| **Vastus medialis** | **0** | **0.011** | **0.002** | 0.083 |
| **Sartorius** | **0.026** | **0.024** | 0.382 | **0.024** |
| **Gracilis** | 0.956 | 1 | 1 | 0.79 |
| **Biceps femoris** | 0.098 | 0.224 | 0.616 | **0.011** |
| **Semitendinosus** | 0.142 | 1 | 0.188 | **0.024** |
| **Semimembranosus** | 0.301 | 0.73 | 0.345 | 0.11 |
| **Adductor magnus** | 0.128 | 0.578 | 0.176 | 0.075 |
| **Adductor longus** | 0.437 | 0.238 | 0.626 | 0.534 |
| **Gluteal** | 0.269 | 0.415 | 0.28 | 0.225 |
| **Tibialis anterior** | 0.182 | 0.354 | 0.151 | 0.497 |
| **Extensor digitorum longus** | 0.851 | 0.835 | 0.602 | 1 |
| **Peroneal** | 0.657 | 0.762 | 0.24 | 0.88 |
| **Tibialis posterior** | **0.008** | 0.077 | **0.041** | **0.023** |
| **Soleus** | 0.929 | 0.675 | 1 | 0.88 |
| **Lateral gastrocnemius** | 0.824 | 1 | 0.492 | 0.59 |
| **Medial Gastrocnemius** | 0.351 | 0.3 | 0.758 | 0.231 |

**Legend: pink highlighted boxes represent statistically significant values (p=<0.05)**
