# Supplementary material for: Muscle magnetic resonance imaging involvement patterns in nemaline myopathies
Source: Ann Clin Transl Neurol. 2023 Jun 2;10(7):1219–29. doi: 10.1002/acn3.51816 (PMC10351659; doi:10.1002/acn3.51816)
Supplement: Supplementary file 4 — Table S4. Involvement score data from source literature by genotype. Where full Mercuri scoring (MS) or equivalent qualitative muscle MRI scoring was available within the source literature, all muscles recorded as having an MS ≥ 2 or equivalent were attributed a score of +1, unless they are commented upon as being notably comparatively spared, in which case they were attributed a score of −1. Muscles with MS of 1 were attributed a score of 0, unless they are remarked upon as notably involved or spared compared to other muscles in which case they were scored as +1 or −1 accordingly. Muscles with an MS of 0 were given a score of −1. Where full MS or equivalent was not available, muscles that have been notably mentioned as involved within the source literature were attributed a score of +1; those mentioned as spared or relatively spared were attributed a score of −1; those muscles not explicitly specified as particularly involved or spared were attributed a score of 0 (note this does not preclude their involvement). Where the source literature references that findings are compatible/classical of a specified muscle involvement pattern—those muscles considered to be involved or spared as part of this referenced pattern were attributed scores of +1 or −1 accordingly. Where the source literature states muscle groups (e.g. quadriceps, hamstring muscles) were diffusely involved/spared, the muscles constituting these groups were attributed a score of +1/−1 accordingly. Red boxes represent scores of +1, blue boxes represent scores of −1, white boxes represent scores of 0. Green rows represent cumulative scores for each muscle according to genotype. Rectus femoris (RF), vastus lateralis (VL), vastus intermedius (VI), vastus medialis (VM), sartorius (SR), adductors (Add), gracilis (GR), semimembranosus (SM), semitendinosus (ST), biceps femoris (BF), tibialis anterior (TA), tibialis posterior (TP), extensor digitorum longus (EDL), peroneal (PER), soleus (SOL), medial gastrocnemiu [file ACN3-10-1219-s003.docx]

**Supplemental table S4: Involvement score data from source literature by genotype**

| **Literature source** | **Gene** | **Age at MRI** | **RF** | **VL** | **VI** | **VM** | **SR** | **GR** | **BF** | **ST** | **SM** | **Add** | **TA** | **EDL** | **PER** | **LGN** | **MGN** | **SOL** | **TP** |
| --- | --- | --- | --- | --- | --- | --- | --- | --- | --- | --- | --- | --- | --- | --- | --- | --- | --- | --- | --- |
| Jungbluth et al. 2004^14^ | *NEB* | 3 | -1 | -1 | -1 | -1 | -1 | -1 | -1 | -1 | -1 | -1 | 1 | 0 | -1 | -1 | -1 | 1 | -1 |
| Jungbluth et al. 2004^14^ | *NEB* | 22 | -1 | -1 | -1 | -1 | -1 | -1 | -1 | -1 | -1 | -1 | 1 | 0 | -1 | -1 | -1 | 1 | -1 |
| Jungbluth et al. 2004^14^ | *NEB* | 7 | -1 | -1 | -1 | -1 | -1 | -1 | -1 | -1 | -1 | -1 | 1 | 0 | -1 | -1 | -1 | 1 | -1 |
| Jungbluth et al. 2004^14^ | *NEB* | 9 | -1 | -1 | -1 | -1 | -1 | -1 | -1 | -1 | -1 | -1 | 1 | 0 | -1 | -1 | -1 | 1 | -1 |
| Jungbluth et al. 2004^14^ | *NEB* | 6 | 1 | 1 | 1 | 0 | 0 | 0 | 1 | 1 | 1 | 0 | 1 | 1 | 1 | 1 | 1 | 1 | 1 |
| Jungbluth et al. 2004^14^ | *NEB* | 6 | 1 | 1 | 1 | 0 | 0 | 0 | 1 | 1 | 1 | 0 | 1 | 1 | 1 | 1 | 1 | 1 | 1 |
| Guttsches et al. 2015^16^ | *NEB* | 41 | 0 | 0 | 1 | 0 | 0 | 0 | 1 | 1 | 1 | -1 | 1 | 0 | 1 | -1 | 1 | -1 | 0 |
| Wunderlich et al. 2018^17^ | *NEB* | 54 | 0 | 0 | 0 | 0 | 1 | 0 | 1 | 1 | 1 | 1 | 0 | 0 | 0 | 0 | 0 | 0 | 0 |
| Scoto et al. 2013^18^ | *NEB* | 6 | -1 | -1 | -1 | -1 | -1 | -1 | -1 | -1 | -1 | -1 | 1 | 1 | 1 | -1 | -1 | 1 | 0 |
| Romero et al. 2009^19^ | *NEB* | 27 | 1 | 1 | 1 | 1 | 1 | 1 | 1 | 1 | 1 | 1 | 1 | 1 | 1 | -1 | -1 | 1 | 1 |
| Quijano-Roy et al. 2012^20^ | *NEB* | N/A | 1 | 1 | 1 | 1 | 1 | 1 | 1 | 1 | 1 | 1 | 1 | 1 | 1 | -1 | -1 | 1 | 1 |
| Wallgren-Patterson et al. 2007^21^ | *NEB* | 29 | -1 | -1 | -1 | -1 | -1 | -1 | -1 | -1 | -1 | -1 | 1 | -1 | -1 | -1 | -1 | -1 | -1 |
| Wallgren-Patterson et al. 2007^21^ | *NEB* | 59 | 0 | 1 | 0 | 0 | 0 | 0 | 0 | 1 | 0 | 0 | 1 | 0 | 0 | 1 | 1 | 1 | 0 |
| Wallgren-Patterson et al. 2007^21^ | *NEB* | 28 | -1 | -1 | -1 | -1 | -1 | -1 | -1 | -1 | -1 | -1 | 1 | -1 | -1 | -1 | -1 | -1 | -1 |
| Wallgren-Patterson et al. 2007^21^ | *NEB* | 30 | -1 | -1 | -1 | -1 | -1 | -1 | -1 | -1 | -1 | -1 | 1 | 1 | 1 | -1 | -1 | -1 | 1 |
| Lehtokari et al. 2011^22^ | *NEB* | 11 | 0 | 0 | 1 | 0 |  |  |  |  |  |  | 1 | 1 | -1 | 1 | 1 | 1 | -1 |
| **Cumulative score** | ***NEB*** |  | **-4** | **-3** | **-2** | **-6** | **-5** | **-6** | **-2** | **-1** | **-2** | **-6** | **15** | **5** | **0** | **-7** | **-5** | **7** | **-2** |
| Castiglioni et al. 2014^23^ | *ACTA1* | 19 | 1 | 1 | 1 | 1 | 1 | 1 | 1 | 1 | 1 | 1 | 1 | 1 | 1 | 1 | 1 | 1 | 1 |
| Jungbluth et al. 2004^14^ | *ACTA1* | 44 | 1 | 1 | 1 | 1 | 1 | 1 | 1 | 1 | 1 | 1 | 1 | 1 | 1 | -1 | -1 | 1 | 1 |
| Jungbluth et al. 2004^14^ | *ACTA1* | 23 | 1 | 1 | 1 | 1 | 1 | 1 | 1 | 1 | 1 | 1 | 1 | 1 | 1 | -1 | -1 | 1 | 1 |
| Jungbluth et al. 2004^14^ | *ACTA1* | 40 | 1 | 1 | 1 | 1 | 1 | 1 | 1 | 1 | 1 | 1 | 1 | 1 | 1 | -1 | -1 | 1 | 1 |
| Jungbluth et al. 2004^14^ | *ACTA1* | 9 | 1 | 1 | 1 | 1 | 1 | 1 | 1 | 1 | 1 | 1 | 1 | 1 | 1 | -1 | -1 | 1 | 1 |
| Zukosky et al. 2015^24^ | *ACTA1* | N/A | 1 | 1 | 1 | 1 | 1 | 1 | 1 | -1 | 1 | -1 | 0 | 0 | 0 | 0 | 0 | 0 | 0 |
| Zukosky et al. 2015^24^ | *ACTA1* | N/A | -1 | 1 | -1 | -1 | 1 | -1 | 1 | 0 | -1 | -1 | 0 | 0 | 0 | 0 | 0 | 0 | 0 |
| Jungbluth et al. 2001^25^ | *ACTA1* | 39 | -1 | -1 | -1 | -1 | 1 | -1 | -1 | -1 | -1 | -1 | 1 | 0 | 1 | -1 | -1 | 1 | 0 |
| Ennis et al. 2015^26^ | *ACTA1* | 0.33 | -1 | -1 | -1 | -1 | -1 | -1 | 1 | 1 | 1 | 1 | 0 | 0 | 0 | 0 | 0 | 0 | 0 |
| Garibaldi et al. 2021^27^ | *ACTA1* | 53 | 0 | 1 | 1 | 1 | 0 | 0 | 1 | 1 | 1 | 1 | 0 | 0 | 0 | 1 | 1 | 0 | 0 |
| Garibaldi et al. 2021^27^ | *ACTA1* | 57 | 0 | 0 | 1 | 0 | 0 | 0 | 0 | 0 | 0 | 0 | 0 | 0 | 0 | 1 | 1 | 0 | 0 |
| O’Grady et al. 2015^28^ | *ACTA1* | 34 | 1 | -1 | 0 | -1 | -1 | -1 | 1 | 1 | 1 | 0 | 0 | 0 | 0 | 0 | 0 | 1 | 0 |
| Lornage et al. 2020^29^ | *ACTA1* | 6 | 0 | 0 | 0 | 0 | 1 | 0 | 0 | 0 | 0 | 0 | 1 | 0 | 0 | 0 | 0 | 1 | 0 |
| Lornage et al. 2020^29^ | *ACTA1* | 9 | 0 | 0 | 0 | 0 | 1 | 0 | 0 | 0 | 0 | 0 | 1 | 0 | 0 | 0 | 0 | 1 | 0 |
| **Cumulative score** | ***ACTA1*** |  | **4** | **5** | **5** | **3** | **8** | **2** | **9** | **6** | **7** | **4** | **8** | **5** | **6** | **-2** | **-2** | **9** | **5** |
| Jarraya et al. 2012^30^ | *TPM2* |  | 1 | 1 | 1 | 1 | -1 | 1 | 1 | 1 | 1 | 0 | 1 | 1 | 1 | 1 | 1 | 1 | 1 |
| Jarraya et al. 2012^30^ | *TPM2* |  | 1 | 1 | 1 | 0 | -1 | 0 | 1 | 0 | 1 | 0 | 1 | 1 | 1 | 1 | 1 | 1 | 1 |
| Jarraya et al. 2012^30^ | *TPM2* |  | 1 | 1 | 0 | 0 | -1 | 0 | 1 | 0 | 1 | 0 | 1 | 1 | 1 | 1 | 1 | 1 | 1 |
| Jarraya et al. 2012^30^ | *TPM2* |  | 1 | 1 | 0 | 0 | -1 | 0 | 1 | 0 | 0 | 0 | 1 | 0 | 1 | 1 | 1 | 1 | 0 |
| Jarraya et al. 2012^30^ | *TPM2* |  | 0 | 1 | 0 | 0 | -1 | 0 |  | 0 | 0 | 0 | 1 | 0 | 1 | 0 | 0 | 1 | 0 |
| Jarraya et al. 2012^30^ | *TPM2* |  | 0 | 0 | 0 | 0 | -1 | 0 |  | 0 | 0 | 0 | 0 | 0 | 1 | 0 | 0 | 1 | 0 |
| Jarraya et al. 2012^30^ | *TPM2* |  | 0 | 0 | 0 | 0 | -1 | 0 |  | 0 | 0 | 0 | 0 | 0 | 0 | 0 | 0 | 1 | 0 |
| Jarraya et al. 2012^30^ | *TPM2* |  | 0 | 0 | 0 | 0 | -1 | 0 |  | 0 | 0 | 0 | 0 | 0 | 0 | 0 | 0 | 1 | 0 |
| Lehtokari et al. 2007^31^ | *TPM2* |  | -1 | 0 | 0 | 0 | -1 | -1 | 1 | 1 | 1 | 1 | 1 | 1 | 1 | 1 | 1 | 1 | 1 |
| Quijano-Roy et al. 2012^20^ | *TPM2* | N/A | 1 | 0 | 0 | 0 | 0 | 0 | 0 | 0 | 0 | 0 | 0 | 1 | 1 | 1 | 0 | 1 | 0 |
| Quijano-Roy et al. 2012^20^ | *TPM2* | N/A | 1 | 0 | 0 | 0 | 0 | 0 | 0 | 0 | 0 | 0 | 0 | 1 | 1 | 1 | 0 | 1 | 0 |
| Quijano-Roy et al. 2012^20^ | *TPM2* | N/A | 1 | 0 | 0 | 0 | 0 | 0 | 0 | 0 | 0 | 0 | 0 | 1 | 1 | 1 | 0 | 1 | 0 |
| Quijano-Roy et al. 2012^20^ | *TPM2* | N/A | 1 | 0 | 0 | 0 | 0 | 0 | 0 | 0 | 0 | 0 | 0 | 1 | 1 | 1 | 0 | 1 | 0 |
| Quijano-Roy et al. 2012^20^ | *TPM2* | N/A | 1 | 0 | 0 | 0 | 0 | 0 | 0 | 0 | 0 | 0 | 0 | 1 | 1 | 1 | 0 | 1 | 0 |
| Quijano-Roy et al. 2012^20^ | *TPM2* | N/A | 0 | 1 | 0 | 0 | 0 | 0 | 0 | 0 | 0 | 0 | 0 | 0 | 0 | 0 | 0 | 1 | 0 |
| Mokbel et al. 2013^32^ | *TPM2* |  | 1 | 1 | 1 | 1 | 0 | 0 | 1 | 0 | 1 | 0 | 0 | 0 | 0 | 0 | 0 | 0 | 0 |
| Tasca et al. 2013^33^ | *TPM2* |  | 0 | 0 | 0 | 0 | 1 | 0 | 0 | 0 | 0 | 0 | 1 | 1 | 1 | 0 | 0 | 0 | 0 |
| Tasca et al. 2013^33^ | *TPM2* |  | 0 | 0 | 0 | 0 | 1 | 0 | 0 | 0 | 0 | 0 | 1 | 1 | 1 | 0 | 0 | 0 | 0 |
| Tasca et al. 2013^33^ | *TPM2* |  | 0 | 0 | 0 | 0 | 1 | 0 | 0 | 0 | 0 | 0 | 1 | 1 | 1 | 0 | 0 | 0 | 0 |
| **Cumulative score** | ***TPM2*** |  | **9** | **7** | **3** | **2** | **-6** | **0** | **6** | **2** | **5** | **1** | **9** | **12** | **15** | **10** | **5** | **15** | **4** |
| Pellerin et al. 2020^34^ | *TNNT1* |  | -1 | -1 | -1 | -1 | -1 | -1 | 0 | 0 | 1 | 1 | 1 | 0 | 0 | -1 | -1 | 1 | 1 |
| Pellerin et al. 2020^34^ | *TNNT1* |  | -1 | -1 | -1 | -1 | -1 | -1 | 0 | 0 | 1 | 1 | 1 | 0 | 0 | -1 | -1 | 1 | 1 |
| Pellerin et al. 2020^34^ | *TNNT1* |  | 0 | 0 | 0 | 0 | 0 | -1 | 0 | 0 | 1 | 1 | -1 | -1 | -1 | -1 | -1 | -1 | -1 |
| Petrucci et al. 2021^35^ | *TNNT1* |  | 0 | 0 | 0 | 0 | 0 | -1 | 1 | 1 | 1 | 1 | 0 | 0 | 0 | 0 | 1 | 1 | 0 |
| Van der Pol et al. 2014^36^ | *TNNT1* |  | -1 | 1 | 1 | 1 | -1 | -1 | -1 | -1 | -1 | 1 | 0 | 0 | 0 | 0 | 0 | 1 | 0 |
| **Cumulative score** | ***TNNT1*** |  | **-3** | **-1** | **-1** | **-1** | **-3** | **-5** | **0** | **0** | **3** | **5** | **1** | **-1** | **-1** | **-3** | **-2** | **3** | **1** |
| Shrekenbach et al. 2014^37^ | *TPM3* | 68 | -1 | -1 | -1 | -1 | -1 | -1 | 1 | -1 | 1 | -1 | 1 | 0 | -1 | -1 | -1 | 1 | 0 |
| Shrekenbach et al. 2014^37^ | *TPM3* | 20 | -1 | -1 | -1 | -1 | -1 | -1 | -1 | -1 | -1 | -1 | -1 | 0 | -1 | -1 | -1 | 1 | 0 |
| Citirak et al. 2014^38^ | *TPM3* | 22 | 1 | -1 | -1 | -1 | -1 | -1 | -1 | -1 | -1 | -1 | 0 | 0 | 0 | 0 | 0 | 0 | 0 |
| Citirak et al. 2014^38^ | *TPM3* | 30 | 1 | -1 | -1 | -1 | -1 | -1 | -1 | -1 | -1 | -1 | 0 | 0 | 0 | 0 | 0 | 0 | 0 |
| Bevilacqua et al. 2022^39^ | *TPM3* | 47 | -1 | 1 | 1 | 1 | 0 | 0 | 1 | 1 | 1 | -1 | 0 | 0 | 0 | 1 | 1 | 1 | 1 |
| Moreno et al. 2020^40^ | *TPM3* | 14 | 0 | 0 | 0 | 0 | 1 | -1 | 1 | 1 | 1 | 0 | 1 | 1 | 1 | -1 | -1 | 1 | 1 |
| **Cumulative score** | ***TPM3*** |  | **-1** | **-3** | **-3** | **-3** | **-3** | **-5** | **0** | **-2** | **0** | **-5** | **1** | **1** | **-1** | **-2** | **-2** | **4** | **2** |
| Yuen et al. 2014^41^ | *LMOD3* |  | 1 | -1 | 1 | 1 | 1 | -1 | 1 | -1 | -1 | 1 | 1 | -1 | 1 | 1 | 1 | 1 | 1 |
| Dofash et al. 2021^42^ | *KLHL40* |  | 0 | 0 | 1 | 0 | 0 | 0 | 1 | 1 | 1 | 1 | 1 | 1 | 1 | 1 | 1 | 1 | 1 |
| Kang et al. 2020^43^ | *KBTBD13* |  | 1 | 1 | 1 | 1 | 0 | -1 | 0 | -1 | 1 | 1 | 1 | 0 | -1 | 0 | 1 | -1 | 0 |
| Garibaldi et al. 2018^44^ | *KBTBD13* |  | 1 | 0 | 0 | 0 | 0 | 0 | 0 | 0 | 0 | 0 | -1 | -1 | -1 | -1 | -1 | -1 | -1 |
| **Cumulative score** | ***KBTBD13*** |  | **2** | **1** | **1** | **1** | **0** | **-1** | **0** | **-1** | **1** | **1** | **0** | **-1** | **-2** | **-1** | **0** | **-2** | **-1** |

Legend: Where full Mercuri scoring (MS) or equivalent qualitative muscle MRI scoring was available within the source literature, all muscles recorded as having a MS ≥2 or equivalent were attributed a score of +1, unless they are commented upon as being notably comparatively spared, in which case they were attributed a score of -1. Muscles with MS of 1 were attributed a score of 0, unless they are remarked upon as notably involved or spared compared to other muscles in which case they were scored as +1 or -1 accordingly. Muscles with a MS of 0 were given a score of -1. Where full MS or equivalent were not available, muscles that have been notably mentioned as involved within the source literature were attributed a score of +1; those mentioned as spared or relatively spared were attributed a score of -1; those muscles not explicitly specified as particularly involved or spared were attributed a score of 0 (note this does not preclude their involvement). Where the source literature references that findings are compatible/classical of a specified muscle involvement pattern – those muscles considered to be involved or spared as part of this referenced pattern were attributed scores of +1 or -1 accordingly. Where the source literature states muscle groups (e.g. quadriceps, hamstring muscles) were diffusely involved/spared, the muscles constituting these groups were attributed a score of +1/-1 accordingly. Red boxes represent scores of +1, blue boxes represent scores of -1, white boxes represent scores of 0. Green rows represent cumulative scores for each muscle according to genotype. Rectus femoris (RF), vastus lateralis (VL), vastus intermedius (VI), vastus medialis (VM), sartorius (SR), adductors (Add), gracilis (GR), semimembranosus (SM), semitendinosus (ST), biceps femoris (BF), tibialis anterior (TA), tibialis posterior (TP), extensor digitorum longus (EDL), peroneal (PER), soleus (SOL), medial gastrocnemius (MGN), lateral gastrocnemius (LGN). Green rows highlight cumulative scores for each genotype
